# Subjective well-being among AIDS orphans in southwest China: the role of school connectedness, peer support, and resilience

**DOI:** 10.1186/s12888-022-03833-2

**Published:** 2022-03-18

**Authors:** Shimin Lai, Junmin Zhou, Xiaohe Xu, Shiying Li, Yuanyi Ji, Shujuan Yang, Wanjie Tang, Jianxin Zhang, Jianjun Jiang, Qiaolan Liu

**Affiliations:** 1 Department of Health Behavior and Social Medicine, West China School of Public Health and West China Fourth Hospital, Sichuan University, Chengdu, China; 2 Department of Medical Affairs, West China Tianfu Hospital, Sichuan University, Chengdu, China; 3 University of Texas at San Antonio, San Antonio, USA; 4 Department of Sociology and Psychology, School of Public Administration, Sichuan University, Chengdu, China; 5 Nosocomial Infection Management Department, West China School of Public Health and West China Fourth Hospital, Sichuan University, Chengdu, Sichuan People’s Republic of China; 6 Centre for Educational and Health Psychology, Sichuan University, Chengdu, China; 7 Department of Maternal, and Child and Adolescent Health, West China School of Public Health and West China Fourth Hospital, Sichuan University, Chengdu, China; 8 Department of Palliative Care, West China School of Public Health and West China Fourth Hospital, Research Center for Palliative Care, West China-PUMC C.C. Chen Institute of Health, Sichuan University, Chengdu, China

**Keywords:** AIDS orphan, Subjective well-being, School connectedness, Resilience, Peer support, Structural equation model

## Abstract

**Background:**

Few studies have explored the health and development of AIDS orphans using the positive youth development (PYD) framework. Grounded in this framework, the main objective of this study is to examine how internal assets (i.e., resilience) and external assets (i.e., school connectedness, peer support) affect subjective well-being among Yi AIDS orphans in the Liangshan Yi Autonomous Prefecture, Sichuan province, China.

**Methods:**

A cross-sectional survey was conducted by interviewing 571 AIDS orphans and 979 non-orphans of Yi ethnic minority from 5th-10th grades. Structural equation models (SEM) were utilized to identify and estimate the direct and indirect effects of internal and external assets on subjective well-being.

**Results:**

The average score of subjective well-being was significantly lower for AIDS orphans than for in non-orphans (*P* < 0.05). Resilience, school connectedness, peer support (number of friends, caring friends), and self-rated physical health had significant and positive direct effects on subjective well-being. In addition, the effects of school connectedness, and peer support on subjective well-being were mediated by resilience.

**Conclusions:**

Positive individual and school-related contextual assets can bolster subjective well-being among AIDS orphans. The design of health intervention programs for AIDS orphans should incorporate these positive development assets.

**Supplementary Information:**

The online version contains supplementary material available at 10.1186/s12888-022-03833-2.

## Introduction

Since the first cases of acquired immunodeficiency syndrome (AIDS) were reported in 1981, human immunodeficiency virus (HIV) infection has become a global pandemic, which has exacted a heavy toll on both adults and children. It has been estimated that 36.3 million people have died from AIDS-related illnesses since the onset of the pandemic [1]. In parallel, globally about 17 million children below 18 years of age have been orphaned by AIDS for losing one or both parents [1, 2]. With reference to China, the number of AIDS orphans was estimated to reach 2.6 million by 2010 [3]. Being an AIDS orphan can have adverse effects on a child's physical and somatic health. As revealed by current research. AIDS orphans are vulnerable to HIV infection and continue to face an increased risk of suffering from poor health and other developmental impediments than non-orphan children, which has been of great concern worldwide.

A growing body of research has suggested that AIDS orphans are more susceptible to internalizing problems, such as depression, anxiety, and suicidal ideation than non-orphans [4–7]. While documenting these pernicious mental health outcomes for AIDS orphans, prior studies relied heavily on deficit models. By contrast, a strength-based approach, known as positive youth development (PYD), has focused instead on internal development assets (e.g., competency and resilience) and external development assets (e.g., social and peer support) to understand and improve children and adolescent health and sociopsychological development [8]. To challenge the limitations of deficit models that focus on youth problems, PYD holistically recognizes, utilizes, and fosters positive outcomes and practices for children and adolescents. The positive outcomes encompass character strengths across different domains of adolescent health, self-efficacy, emotional competence, and social development. The practices of PYD emphasize a wide array of positive contexts including, but not limited to, peer group, family, school, and the larger culture and policy environment. One of the critical aspects of the PYD framework for AIDS orphans is to provide the positive developmental nutrients that AIDS orphans need to foster their subjective well-being, which refers to AIDS orphan's cognitive and affective appraisal across different domains of their lives and emotional experience [9, 10]. Informed by the PYD framework, it is argued that both internal and external development assets can work in tandem to promote and improve subjective well-being for AIDS orphans.

Since school-age AIDS orphans spend most of their time in school, factors such as school connectedness and peer support are deemed important external development assets for subjective well-being [11]. School connectedness connotes the students' perceptions about their personal inclusion, acceptance, and support in the social environment of the school [12–14]. Prior research indicates that children and adolescents with higher levels of school connectedness are less likely to exhibit poor mental health symptoms such as depression, anxiety, suicidal ideation, and risk behavior. By the same token, children and adolescents with higher levels of school connectedness are more likely to report a supportive and fair school environment that fosters their emotional well-being [15].

Peer support, defined as the process of giving and receiving emotional and practical assistance, sharing knowledge, teaching and learning life skills, and connecting people with resources and opportunities within a network of friends or peers, is another important positive development asset for children and adolescents' subjective well-being [16, 17]. Resembling the positive effects of school connectedness on mental health, students with higher levels of peer support are less likely to exhibit depression and anxiety symptoms [18]. As demonstrated by a cluster-randomized trial study, peer support decreased AIDS orphans' depression, anxiety, and anger, and at the same time, improved AIDS orphans' psychosocial well-being [19].

Resilience has recently come to light as one of the most salient internal development assets and individual strengths that plays a vital role in recovering and maintaining a well-functioning adaptive system in the context of trauma, tragedy, and other adverse life events. Resilience can be divided into three dimensions: tenacity (calm, steadfast, responsive, and in control), strength (resilience after setbacks can not only recover, but also develop and grow), and optimism (obtaining and retaining confidence in overcoming adversity, viewing things from a positive perspective, etc.) [20, 21]. A substantial body of literature has documented the significance of resilience in ameliorating children and adolescents' responses to a wide range of adversities, including poverty, parental illness/death, maltreatment, and disastrous life events [22–24]. One study that reviewed recent research findings indicated that resilience could improve an array of poor mental health outcomes, including depression, post-traumatic stress disorder, loneliness, and/or risk-taking behavior in children affected by AIDS [25]. Prior research has also shown that school connectedness and peer support can enhance resilience in disadvantaged children [26]. Therefore, resilience can serve as an internal development asset that mediates the associations between school connectedness/peer support and subjective well-being.

Moreover, unhealthy physical conditions can hinder children and adolescents' participation in daily learning and activities, which can in turn reduce social connections and life satisfaction. On the other hand, healthy physical conditions can be regarded as a positive health asset that is beyond the mere absence of disease for subjective well-being [27]. Such a positive health asset approach can shed newer light on AIDS orphans' subjective well-being.

In summary, few studies have explored the health and development of AIDS orphans using the PYD framework. Grounded in this framework, the present study examines the subjective well-being of AIDS orphans in the Liangshan Yi Autonomous Prefecture, located in the southwestern part of Sichuan province, China. AIDS orphans in this study refer to children and adolescents under the age of 18 who have lost one or both parents due to AIDS related causes. The vicious circle of intravenous drug use and the high prevalence of HIV/AIDS, coupled with severe economic underdevelopment and chronic poverty, have resulted in a large number of deaths of young adults, thus leaving behind approximately 25,000 AIDS orphans [28]. In addition to facing stigma, life stress, and economic hardship, the lives of AIDS orphans have become unstable due to the lack of care and economic support. To better understand how these devastating disadvantages have shaped AIDS orphans' lives in this minority region, the primary goal of the present study is to examine the effects of the external (school connectedness and peer support) and internal (resilience) development assets on subjective well-being among AIDS orphans of Yi ethnic minority. The secondary goal of this study is to investigate the mediating role of resilience on the associations between school connectedness/peer support and subjective well-being. Last but not least, this study also aims to help develop a positive intervention framework based on the PYD approach to subjective well-being among AIDS orphans of Yi ethnic minority.

## Methods

### Study design and participants

A cross-sectional survey on the well-being of Yi children and adolescents was conducted from September 20 to October 10, 2018. Among the 17 counties in the Liangshan Yi Autonomous Prefecture, Sichuan province, China, four counties that have been seriously affected by HIV/AIDS were selected. Four schools, one from each county, were identified and selected for this study, including one primary school, one middle school, and two schools with mixed grades ranging from 1^st^ grade to 10^th^ grade. Based on the information available through the school management system, AIDS orphans were selected as the study subjects. To ensure accuracy, all AIDS orphans were verified by the school teachers or administrators who were familiar with the student participant's family circumstances. In addition, 1–3 non-orphans were randomly selected from the orphans' classes in each selected school to compose a quasi-comparison group. Both the participants and their guardians were provided with both oral and written consent prior to the study. Before the survey was conducted, all investigators received professional training. By virtual of their professional qualifications, several investigators served as counselors for the student participants throughout the data selection process. In total, 1,912 students ranging from 1^st^ grade to 10^th^ grade were recruited. Because of their limited literacy and proficiency, the students in 1^st^-4^th^ grades were excluded from this study. As a result, 1,550 participants with an average age of 14.3 years [standard deviation (SD): 1.77 years] from 5^th^ grade to 10^th^ grade constitute an analytic subsample for the present study. Out of the 1,550 Yi adolescents, 571 were AIDS orphans and 979 were non-orphans.

### Instruments

#### Subjective well-being

Subjective well-being was measured by the Index of Well-being [29], consisting of two parts: (1) the 8-item Index of General Affect Scale and (2) the single-item Life Satisfaction Questionnaire. All items were rated on a 7-point Likert scale. The final composite measure was the summed-score index of the two parts with the weight of 1:1.1, ranging from 2.1 (extremely unhappy) to 14.7 (extremely happy). This scale has been validated in the Chinese context [30]. The Cronbach's alpha coefficient of the subjective well-being index in this study was 0.84, suggesting good internal consistency and reliability.

#### School connectedness

The School Connectedness Scale came from the Resilience Youth Development Module of the California Healthy Kids Survey. There were 5 items [31]: (1) I feel close to people at this school, (2) I am happy to be at this school, (3) I feel like I am part of this school, (4) The teachers at this school treat students fairly, and (5) I feel safe in my school. Participants were asked to choose the option that best described them on a 5-point Likert scale, ranging from 1 = strongly disagree to 5 = strongly agree. Higher scores in the scale indicated higher levels of school connectedness. This scale has been used and validated in the Chinese context [32]. The Cronbach's alpha coefficient of the scale was 0.72.

#### Peer support

Two items from the Social Support Rating Scale were utilized to represent peer support [33]. Participants were first asked: "How many good friends (or classmates) do you have?” The four-point response options were 1 = none, 2 = 1–2, 3 = 3–5, and 4 = 6 and above. They were then asked: "Do your classmates (or friends) care about you?” Responses were captured on 4-point scale with 1 = indifference, 2 = occasionally, 3 = moderately often, and 4 = very often.

#### Self-rated physical health

In addition, a single item was included to measure self-rated physical health. Participants were asked: "How do you feel about your physical health?” The five-point Likert response options were: 1 = very unhealthy, 2 = unhealthy, 3 = moderately healthy, 4 = healthy, and 5 = very healthy.

#### Resilience

The Connor-Davidson Resilience Scale developed by Connor and Davidson include 25 items, for example, "I can adapt to change" and "I make my best effort no matter what". This scale was adapted and divided into three dimensions: (1) tenacity, (2) strength, and (3) optimism. The items were rated on a 5-point scale (0 = never to 4 = all the time) and summed to form an index variable [34], with higher scores indicating better resilience [35]. This scale has been used and validated in the Chinese context as well [36]. The Cronbach's alpha coefficient was 0.90.

#### Other sociodemographic characteristics

Demographic characteristics included age (years) and gender (dummy-coded). The housing condition was used to approximate family economic status. The mud-brick house, brick house, and storied house indicated poor, medium, and good family economic status, respectively.

### Data analysis

For descriptive statistical analysis, percentages, means and standard deviations (SD) were calculated and reported. The differences between AIDS orphans and non-orphans in sociodemographic characteristics, subjective well-being, school connectedness, peer support (number of friends and caring friends), resilience and self-rated physical health were analyzed. The partial correlation coefficients among subjective well-being, school connectedness, peer support, resilience, housing, and self-rated physical health were examined, adjusting for age and gender among AIDS orphans.

One structural equation model (SEM) with maximum likelihood estimation was estimated to test both the direct and indirect effects of the exogenous variables on subjective well-being among AIDS orphans. The mediating effects of resilience on subjective well-being was tested using the bootstrapping method. The following indicators were used to evaluate the goodness-of-fit of the model: the goodness-of-fit index (GFI) of 0.90 or better; the comparative fit index (CFI) of 0.90 or better; the normed fit index (NFI) of 0.90 or better; the adjusted goodness-of-fit index (AGFI) of 0.90 or better; and the root mean square error of approximation (RMSEA) of less than 0.08 [37]. The statistical analyses were performed using SPSS 22.0 (IBM, Armonk, NY, USA) and AMOS 21.0 (IBM, Armonk, NY, United States). A *P*-value (two-sided) ≤ 0.05 was considered statistically significant.

## Result

As shown in Table 1, the average scores of subjective well-being and resilience were significantly lower for AIDS orphans than for non-orphans (*P* < 0.05). With reference to peer support, 44.5% of AIDS orphans reportedly had more than 6 friends and 31.3% of orphans reported being cared for very often by friends, both of which were lower than non-orphans, 59.2% and 40.5%, respectively. Additionally, nearly 35% of AIDS orphans reported being very healthy, which is also lower than non-orphans (40.9%). Lastly, AIDS orphans reported significantly poorer housing conditions than non-orphans.

**Table 1:** Comparison between AIDS orphans and non-orphans

**Variable**	**Non-orphan (*n* = 979)**	**Orphan (*n* = 571)**	**t/**${\chi }^{2}$	***P-*Value**
	M ± SD)/n(%)	(M ± SD)/n(%)		
**Age**	14.34 ± 1.735	14.29 ± 1.831	0.512	0.609
**Gender**				
Male	471(49.4)	259(48.5)	0.783	0.376
Female	483(50.6)	292(51.5)		
**Housing**				
Mud-brick house	284(29.2)	207(36.4)	8.658	0.013
Brick house	598(61.4)	315(54.4)		
Storied house	92(9.4)	47(8.3)		
**Number of friends**				
None	15(1.5)	15(2.6)	31.938	< 0.001
1–2	150(15.4)	123(21.6)		
3–5	234(24.0)	178(31.3)		
≥ 6	578(59.2)	253(44.5)		
**Caring friends**				
Indifference	28(2.9)	23(4.0)	13.581	0.004
Occasionally	169(17.3)	117(20.6)		
Moderately often	384(39.3)	250(44.0)		
Very often	395(40.5)	178(31.3)		
**Self-rated physical health**				
Very unhealthy	19(1.9)	6(1.1)	10.258	0.036
Unhealthy	67(6.8)	52(9.1)		
Moderate	216(22.1)	150(26.3)		
Healthy	277(28.3)	164(28.6)		
Very healthy	400(40.9)	199(34.9)		
**Resilience**	50.03 ± 15.93	47.03 ± 15.77	3.588	< 0.001
**School connectedness**	18.53 ± 3.56	18.43 ± 3.55	0.555	0.579
**SWB**	10.72 ± 2.26	10.04 ± 2.40	5.582	< 0.001

^a^SWB represents subjective well-being

The adjusted partial correlation coefficients of the study variables are presented in Table 2. It is observed that all study variables are statistically correlated with the exception of housing condition and self-rated physical health. While housing condition was significantly correlated with life satisfaction, self-rated physical health was significantly correlated with school connectedness, one dimension of resilience, the index of general affect, and life satisfaction.

**Table 2:** Partial correlation analysis among AIDS orphans (*n* = 571)

Variable	1	2	3	4	5	6	7	8	9	10	M	SD
1.Tenacity^a^	1										24.42	9.12
2.Strength^a^	0.745^c^	1									16.15	5.69
3.Optimism^a^	0.561^c^	0.565^c^	1								6.57	2.83
4.School connectedness	0.273^c^	0.252^c^	0.160^c^	1							18.46	3.48
5.Housing	-0.007	-0.012	0.064	-0.027	1						1.72	0.60
6.Number of friends	0.110^c^	0.147^c^	0.139^c^	0.125^c^	0.013	1					3.18	0.85
7.Caring friends	0.123^c^	0.146^c^	0.106^c^	0.190^c^	0.071	0.249^c^	1				3.02	0.82
8.Self-rated physical health	-0.005	0.058	0.089^c^	0.166^c^	0.052	0.116^c^	0.203^c^	1			3.89	1.02
9.Index of general affect^b^	0.134^c^	0.183^c^	0.160^c^	0.361^c^	0.044	0.111^c^	0.223^c^	0.243^c^	1		5.38	1.61
10.Life satisfaction^b^	0.244^c^	0.251^c^	0.197^c^	0.365^c^	0.097^c^	0.280^c^	0.272^c^	0.272^c^	0.537^c^	1	4.71	1.08

^a^Subscales of the resilience, ^b^Subscales of the SWB

^c^Significance at the 0.05 level (2-tailed); adjusting for age and gender

**Fig. 1:**
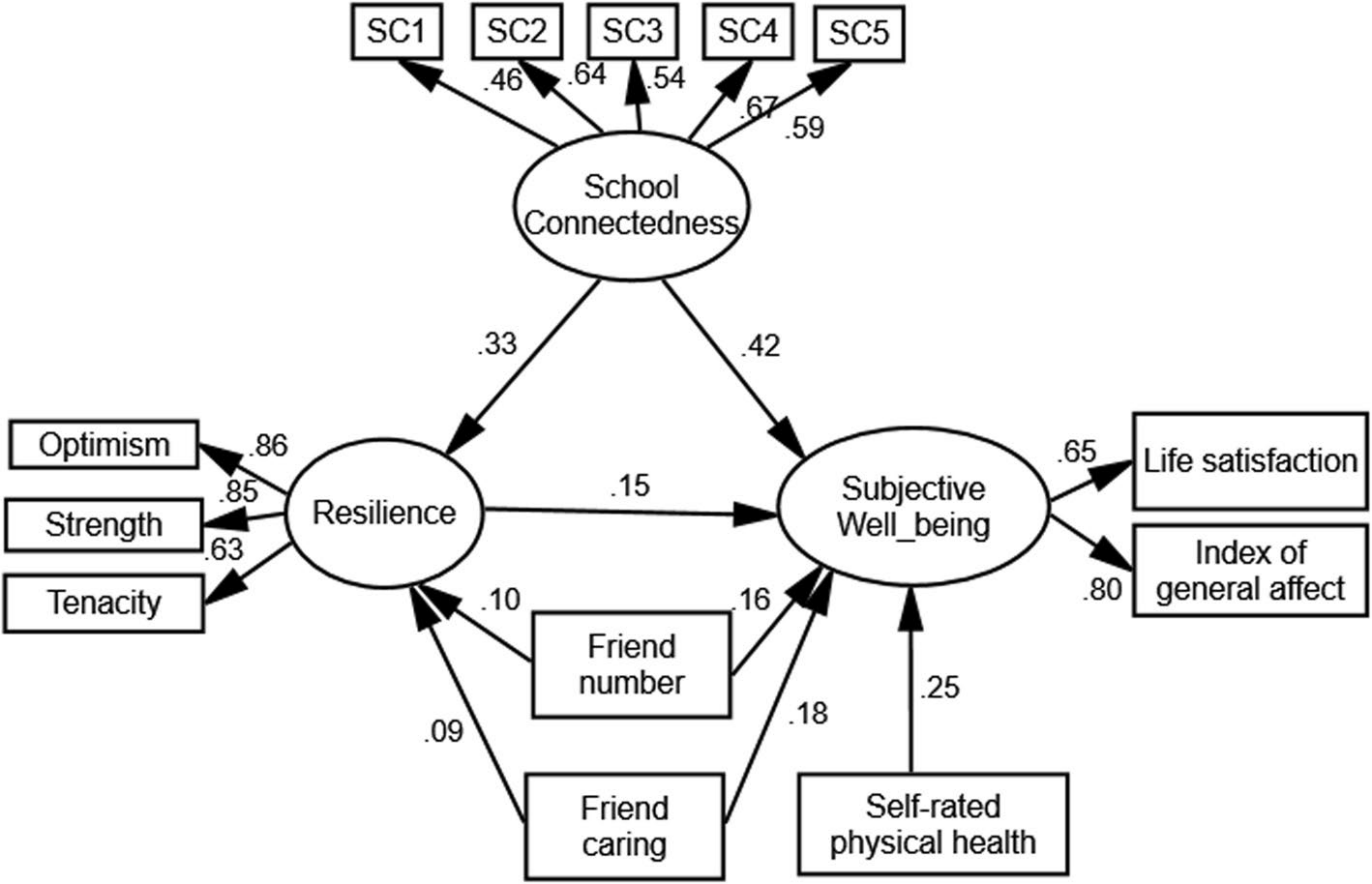
School connectedness, peer support, resilience and subjective well-being: Standardized SEM coefficients for AIDS orphans (*n* = 571). Notes: SC1-SC5 represent 5 items of the school connectedness scale

Figure 1 displays the SEM results for AIDS orphans. The model fit was within the acceptable ranges with AGFI = 0.936, GFI = 0.958, CFI = 0.940, NFI = 0.908, and RMSEA = 0.054, and the *P*-value associated with each path coefficient was less than 0.05, the exact times of the bootstrap method was 5000. Table 3 reports both direct and indirect effects. The results indicate that school connectedness, peer support in terms of the number of friends and caring friends, and self- rated physical health had significant and direct effects on subjective well-being. In addition, the effects of school connectedness, number of friends, and caring friends on subjective well-being were mediated by resilience (indirect effects are 0.049, 0.015, and 0.013, respectively; *P* < 0.05). These mediation effects account for 10.5% of the total effect of school connectedness, 8.3% of the total effect of the number of friends, and 6.7% of the total effect of caring friends on subjective well-being, respectively. The same model was also estimated for non-orphans (Appendix Table 1 and Appendix Fig. 1). Except for the number of friends, other associations were consistent with those for AIDS orphans.

**Table 3:** The standardized effects of school connectedness, peer support, and resilience on subjective well-being among AIDS orphans (*n* = 571)

**Pathway**^b^	**Estimated effect**	**95%CI of estimated effect**
**Total effects**		
School connectedness → SWB	0.467^a^	(0.331, 0.587)
Number of friends → SWB	0.179^a^	(0.070, 0.275)
Caring friends → SWB	0.194^a^	(0.102, 0.305)
**Direct effects**		
School connectedness → SWB	0.418^a^	(0.270, 0.548)
Number of friends → SWB	0.164^a^	(0.057, 0.262)
Caring friends → SWB	0.181^a^	(0.087, 0.291)
Self-rated physical health → SWB	0.247^a^	(0.148, 0.340)
Resilience → SWB	0.146^a^	(0.027, 0.253)
School connectedness → Resilience	0.334^a^	(0.239, 0.432)
Number of friends → Resilience	0.105^a^	(0.007, 0.198)
Caring friends → Resilience	0.089^a^	(0.004, 0.181)
**Indirect effects**		
School connectedness → Resilience → SWB	0.049^a^	(0.012, 0.088)
Number of friends → Resilience → SWB	0.015^a^	(0.001, 0.043)
Caring friends → Resilience → SWB	0.013^a^	(0.001, 0.038)

^a^Significance at the 0.05 level (2-tailed)

^b^SWB represents subjective well-being

## Discussion

This study has provided a new direction for research on the positive development of AIDS orphans. As expected, this study found that the sheer amount of developmental assets AIDS orphans experienced, including school connectedness, peer support, resilience, and better physical health, was appreciably and positively associated with improved subjective well-being. Such findings are consistent with the PYD framework in that AIDS orphans could obtain positive outcome,such as improved subjective well-being, if their individual strengths (i.e., internal assets) are well aligned with supportive ecological assets (i.e., external assets). This means that as a central construct in the framework of developmental assets and mental health promotion, subjective well-being has considerable implications for positive adolescent outcomes in their transition to young adulthood [8, 38, 39]. As such, a high level of subjective well-being among AIDS orphans signals healthy social, emotional, and mental functioning. To translate these research findings into public health practice, practitioners are urged to actively identify key internal and external influencing factors of subjective well-being from the perspective of variability and operability in order to promote AIDS orphans' subjective well-being. Stated differently, the findings from this study suggest that to develop and implement culturally competent intervention programs to improve subjective well-being for AIDS orphans of Yi ethnic minority, not only should we consider the resources internal to AIDS orphans, but also the role of the external environment.

To cultivate ecological assets, this study found that school connectedness, as a crucial external asset, positively affected AIDS orphans' subjective well-being. School connectedness is the perception developed by students about the care and respect they received from their teachers and other adults in the school context. A higher level of school connectedness would reflect positive attitudes toward school, a strong sense of belonging, and a positive connection with adults and peers. This sense of belonging can help reduce AIDS orphans' psychological distress [13, 15]. Therefore, fostering school connectedness has been considered as a practical evidence-based strategy for mental health promotion among children and adolescents [26, 40]. By law, children and adolescents in China (aged 6–17) can obtain free education in public schools for nine years supported by the Chinese government via Compulsory Education Law. Even under the dual threats of the HIV/AIDS epidemic and poverty, school-age AIDS orphans of Yi ethnic minority will continue to attend schools. As such, school environmental intervention is feasible in the context of improving AIDS orphans' subjective well-being.

As anticipated, peer support surfaced as another external asset that significantly bolstered AIDS orphans' subjective well-being. Results from this study showed that having more friends, especially caring friends, might imply frequent information sharing and emotional support AIDS orphans elicited from their peer networks. Such intangible peer support could meet basic psychological needs and make up for the lack of familial support for AIDS orphans, which underscored the importance of peer support as a positive development asset for children and adolescents' subjective well-being.

As an internal development asset, results pertaining to resilience are in line with the prior literature. That is, resilience could play a vital role in strengthening subjective well-being even when AIDS orphans encountered a significant amount of adversity and stress [41]. Put differently, developing and implementing effective intervention strategies to increase resilience, especially maintaining an optimistic attitude when facing adversity and stress, can help boost AIDS orphans' capacity to overcome the detrimental effects of adversity, thereby improving psychosocial well-being [42]. In addition to the direct effect of resilience on subjective well-being for AIDS orphans, this study also established the mediating role of resilience. This means that school connectedness and peer support could elevate AIDS orphans' levels of resilience, which could in turn bolster subjective well-being. Since the level of resilience was much lower for AIDS orphans than for non-orphans, the resilience-based intervention strategy is recommended in efforts to improve AIDS orphans' subjective well-being [43, 44]. Resiliency-based intervention strategies can be developed in the form of health education and counseling that centers on AIDS orphans' emotional regulation, stress management, interpersonal and problem solving skills and a better understanding of adversity [45]. Additionally, group-based boding activities are recommended for the schools attended by AIDS orphans. Such activities can be used to create a harmonious atmosphere and cultivate the ties between teachers, peers, and AIDS orphans.

For children and adolescents, self-rated physical health reflects self-perceived health conditions (e.g., fatigue, stomachache, headache or having a physical disability). Under the PYD framework, self-reported health can be viewed as health asset. As such, good healthy conditions can be positively associated with subjective well-being [27], which is confirmed by our study findings. This could be due to the fact that AIDS orphans who reported better physical health might be more likely to participate in physical activities and better integrated into their peer group(s). Therefore, physical activity can be a positive factor for subjective well-being as well [46]. Unfortunately, this study did not obtain information about AIDS orphans' physical activity. Thus, the effect of self-rated physical health can be viewed as an indirect measure of physical activity on subjective well-being.

### Strengths and limitations

Several research limitations should be recognized. First, this study utilized a cross-sectional survey. As such, it is impossible to draw any causal conclusions regarding the associations between school connectedness, peer support, or resilience, and subjective well-being among AIDS orphans. Longitudinal studies are needed to establish possible causal relationships in future research. Second, all variables used in this study were self-reported, which might be subject to a certain degree of self-report bias. However, it is important to note that our SEM analysis has corrected some of the measurement errors (i.e., self-report bias) by design. Finally, several important measures that are associated with AIDS orphans' subjective well-being, such as self-esteem, relationship with the caregiver, and physical activity, were not included in the survey. Future research should incorporate these factors as developmental assets and link them to AIDS orphans' subjective well-being.

Despite these limitations, there are some strengths to this study. The most notable strength is the contribution to the study of AIDS orphans' subjective well-being from a positive development perspective. We venture to suggest that the PYD framework has the potential to become a leading theoretical framework in studying AIDS orphans. Additionally, the sample size of this study is reasonably large, and the findings are generalizable to AIDS orphans of Yi ethnic minority in the region. Finally, this study has laid a compelling backdrop for (1) future intervention work that can help AIDS orphans thrive by increasing both external assets (e.g., school connectedness, peer support) and internal assets (e.g., resilience) and (2) mobilizing and orchestrating government and public efforts and resources to improve AIDS orphans' and non-orphan adolescents' subjective well-being among ethnic minority populations in China.

## Conclusions

The PYD framework is essential for scholars and practitioners to identify school-related contextual and individual assets that can bolster AIDS orphans' subjective well-being. Practitioners are strongly encouraged to offer health promotion and health education programs so that school-age children and adolescents, especially AIDS orphans, can receive the necessary training to improve their health-related, problem-solving, and coping skills. Meanwhile, school administrators and teachers can utilize these training opportunities to promote subjective well-being for AIDS orphans.

## Supplementary Information

**Additional file 1.**

**Additional file 2.**

## Data Availability

The datasets generated and/or analyzed during the current study are not publicly available due to limitations of ethical approval involving the patient data and anonymity but are available from the corresponding author upon reasonable request.
