# Supplementary material for: Subjective well-being among AIDS orphans in southwest China: the role of school connectedness, peer support, and resilience
Source: BMC Psychiatry. 2022 Mar 18;22:197. doi: 10.1186/s12888-022-03833-2 (PMC8933895; doi:10.1186/s12888-022-03833-2)
Supplement: Supplementary file 1 — Additional file 1. [file 12888_2022_3833_MOESM1_ESM.docx]

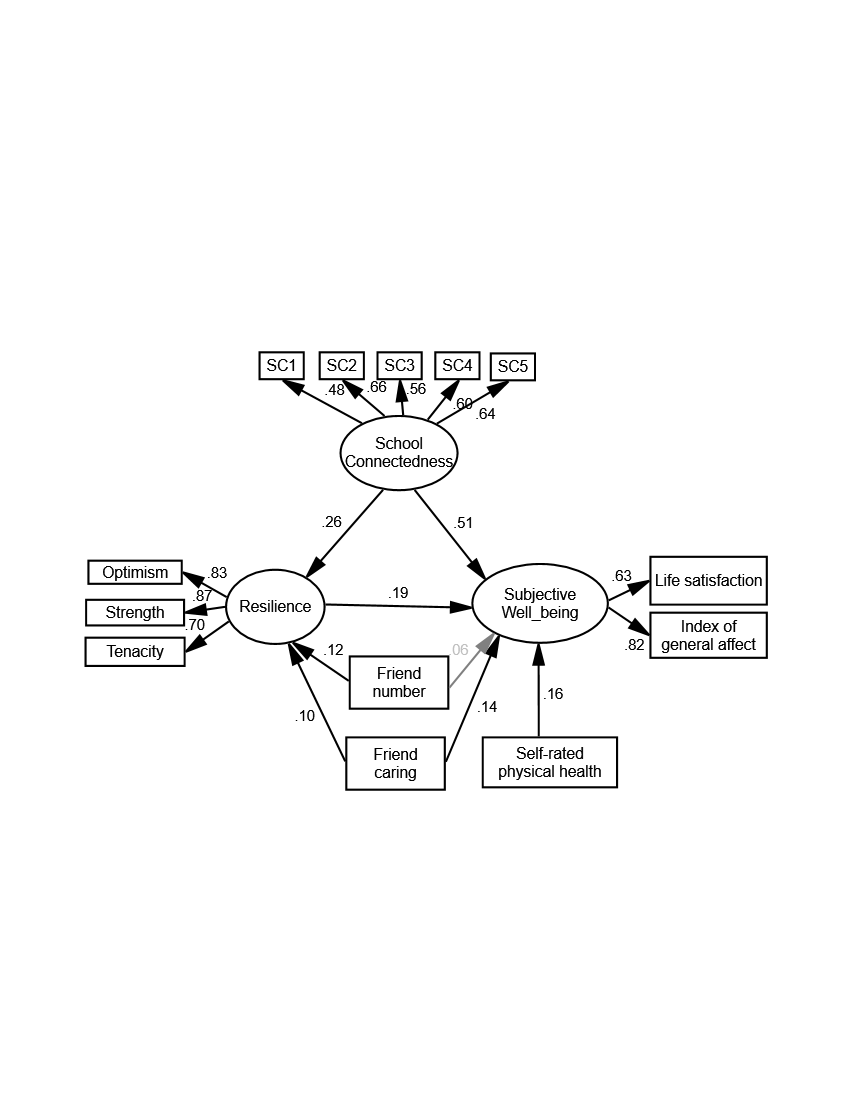


Appendix Figure 1 School connectedness, peer support, resilience and subjective well-being: Standardized SEM coefficients for non-orphans (n=979)

Notes: SC1-SC5 represent 5 items of the school connectedness scale. The gray line means not statistically significant.
