# Supplementary material for: Subjective well-being among AIDS orphans in southwest China: the role of school connectedness, peer support, and resilience
Source: BMC Psychiatry. 2022 Mar 18;22:197. doi: 10.1186/s12888-022-03833-2 (PMC8933895; doi:10.1186/s12888-022-03833-2)
Supplement: Supplementary file 2 — Additional file 2. [file 12888_2022_3833_MOESM2_ESM.docx]

Appendix Table1. The standardized effects of school connectedness, peer support, and resilience on subjective well-being among non-orphans (n=979)

| Pathway^#^ | Estimated effect | 95%CI of estimated effect |
| --- | --- | --- |
| **Total effects** |  |  |
| School connectedness→SWB | 0.555^*^ | （0.457, 0.646） |
| Number of friends→SWB | 0.079^*^ | （0.001, 0.149） |
| Caring friends→SWB | 0.156^*^ | （0.082, 0.224） |
| **Direct effects** |  |  |
| School connectedness→SWB | 0.506^*^ | （0.410, 0.605） |
| Number of friends→SWB | 0.056 | （-0.021, 0.126） |
| Caring friends→SWB | 0.137^*^ | （0.063, 0.205） |
| Self-rated physical health→SWB | 0.162^*^ | （0.092, 0.238） |
| Resilience→SWB | 0.189^*^ | （0.102, 0.272） |
| School connectedness→Resilience | 0.257^*^ | （0.169, 0.345） |
| Number of friends→Resilience | 0.120^*^ | （0.047, 0.198） |
| Caring friend→Resilience | 0.099^*^ | （0.030, 0.173） |
| **Indirect effects** |  |  |
| School connectedness→Resilience→SWB | 0.049^*^ | （0.027, 0.080） |
| Number of friends→Resilience→SWB | 0.023^*^ | （0.008, 0.044） |
| Caring friends→Resilience→SWB | 0.019^*^ | （0.006, 0.041） |

*Signiﬁcance at the 0.05 level (2-tailed).

^#^SWB represents subjective well-being.
